# The influence of weather and urban environment characteristics on upper respiratory tract infections: a systematic review

**DOI:** 10.3389/fpubh.2025.1487125

**Published:** 2025-02-10

**Authors:** Henna Hyrkäs-Palmu, Timo T. Hugg, Jouni J. K. Jaakkola, Tiina M. Ikäheimo

**Affiliations:** ^1^Center for Environmental and Respiratory Health Research, Research Unit of Population Health, University of Oulu, Oulu, Finland; ^2^Medical Research Center, Oulu University Hospital, Oulu, Finland; ^3^Department of Community Medicine, UiT the Arctic University of Norway, Tromsø, Norway

**Keywords:** respiratory tract infection, temperature, weather, urban built environment, population density

## Abstract

**Background:**

Weather can independently affect the occurrence of respiratory tract infections (RTIs) in urban areas. Built environments of cities could further modify exposure to weather and consequently the risk of RTIs, but their combined effects on infections are not known.

**Objectives:**

Our aim was to synthesize evidence of the influence of weather on RTIs in urban areas and to examine whether urban built environments are associated with both weather and RTIs.

**Methods:**

A systematic search of Scopus, PubMed, and Web of Science databases was conducted on 9th of August 2022 following PRISMA guidelines. Studies were included in the review based on predefined criteria by screening 5,789 articles and reviewing reference lists of relevant studies. The quality of the studies was assessed using the AXIS appraisal tool, and the results analyzed by narrative synthesis.

**Results:**

Twenty-one eligible studies focusing on COVID-19 and influenza transmissions, were included in the review. All studies were register based ecological studies by design. Low temperature (11/19 studies) was most often associated with increased risk of RTI. Humidity showed either negative (5/14 studies), positive (3/14 studies) or no (6/14 studies) relation with RTIs. The association between wind and solar radiation on infections was inconclusive. Population density was positively associated with RTIs (14/15 studies).

**Conclusions:**

Our review shows that exposure to low temperature increases the occurrence of RTIs in urban areas, and where also high population density increases the infection risk. The study highlights the need to further assess the relationship between built environment characteristics, weather, and RTIs.

## Introduction

A respiratory tract infection (RTI) is a medical condition affecting either the upper, lower or both parts of the respiratory system. Upper RTIs include diseases such as the common cold, sinusitis, pharyngitis and laryngitis. Furthermore, lower RTIs include bronchitis, bronchiolitis, and pneumonia (1, 2). In 2021 the global number of new episodes of upper RTIs was 12.8 billion and accounted for 19,600 deaths (1). Concerning lower RTIs, an estimated 344 million incident episodes and 2.18 million deaths occurred in 2021 (2). COVID-19 is an infectious disease caused by the SARS-CoV-2 virus and can affect both the upper and lower respiratory tract and with either a mild or severe course of the illness. The impact of the COVID-19 infections has also been substantial, and 15.9 million deaths related to the pandemic were registered between 2020 and 2021 (3). In summary, RTIs are a significant global public health concern.

Urban design has been shown to influence health in many ways (4) and can also affect the spread and occurrence of RTIs. For example, suggested important drivers for the spread of COVID-19 infections in urban areas are population density, land use, transportation, mobility, housing conditions, demographic, socio-economic, and health-related factors (5). Also, weather parameters, such as temperature, humidity, wind, and solar radiation, may independently, or in interaction with each other relate to contracting a RTI (6). This would occur because weather could influence the stability of viral pathogens in droplets and aerosols of air or influence transmission through altering the host airway defense, as well as behavior of humans (degree of outdoor exposure) (7).

Previous studies suggest that both temperature and humidity of urban areas affect the risk of contracting RTI (6, 8). Wind has also been shown to be associated with the occurrence of RTIs which could occur either through increasing the spread or diluting the number of airborne pathogens in the environment (9). Low wind speed in cities worsens air pollution (10) and could also indirectly affect the risk of contracting RTIs (11). UV-radiation could also increase the occurrence of RTIs, but the available scientific evidence is scarce (8). To our knowledge, empirical evidence of the exposures of weather on the occurrence of RTIs is either inconclusive (temperature, humidity) or largely lacking (wind, UV-radiation).

Urban built environments (such as buildings, streets and roads and public spaces) can further influence how people are exposed to weather (10) and potentially to microbial agents causing RTIs. For example, the ambient temperature in cities is affected both by characteristics of the built environment and human activity. Urban built structures, such as the height and shape of buildings, their materials and orientation toward the sun, as well as roads and pavements contribute to heat radiation and moisture evaporation (12). In addition, parks and overall greenness can significantly affect evaporative cooling and overall temperature of cities (13, 14). Because of both the anthropogenic activities and urban built environments, city centers are warmer than the surrounding areas. Traditionally, this urban heat island (UHI) effect has been defined as the difference in near-surface air temperature between the urban core and its rural hinterland (10, 15). The flow of wind of urban areas can also be influenced by the density, height/width, location and even shapes of buildings, as well as the arrangement of streets in cities and structures produced by nature (16, 17). Also, higher temperatures of UHIs can create vertical temperature gradients that influence the mixing of air and affect wind patterns (18). The surrounding environment of cities (mountain basins, valleys) can further influence the wind flow to and away from the city by controlling wind direction (10).

To our knowledge research is lacking on the potential influence of heterogeneity in exposure to weather within urban districts and neighborhoods that are affected by urban built environments, and that could also relate to the occurrence of respiratory infections. Therefore, the aim of this review was to summarize the current evidence of how (1) weather influences the incidence of RTIs in urban environments and how (2) built environment characteristics independently, or in combination with weather, are associated with the incidence of RTIs.

## Methods

This review has not been registered in any database. Our study follows the Preferred Items for Systematic Reviews and Meta-analysis (PRISMA) 2020 methodological guidelines for reporting systematic reviews (19) and included as Supplements 1, 2.

### Search strategy

**Figure 1:**
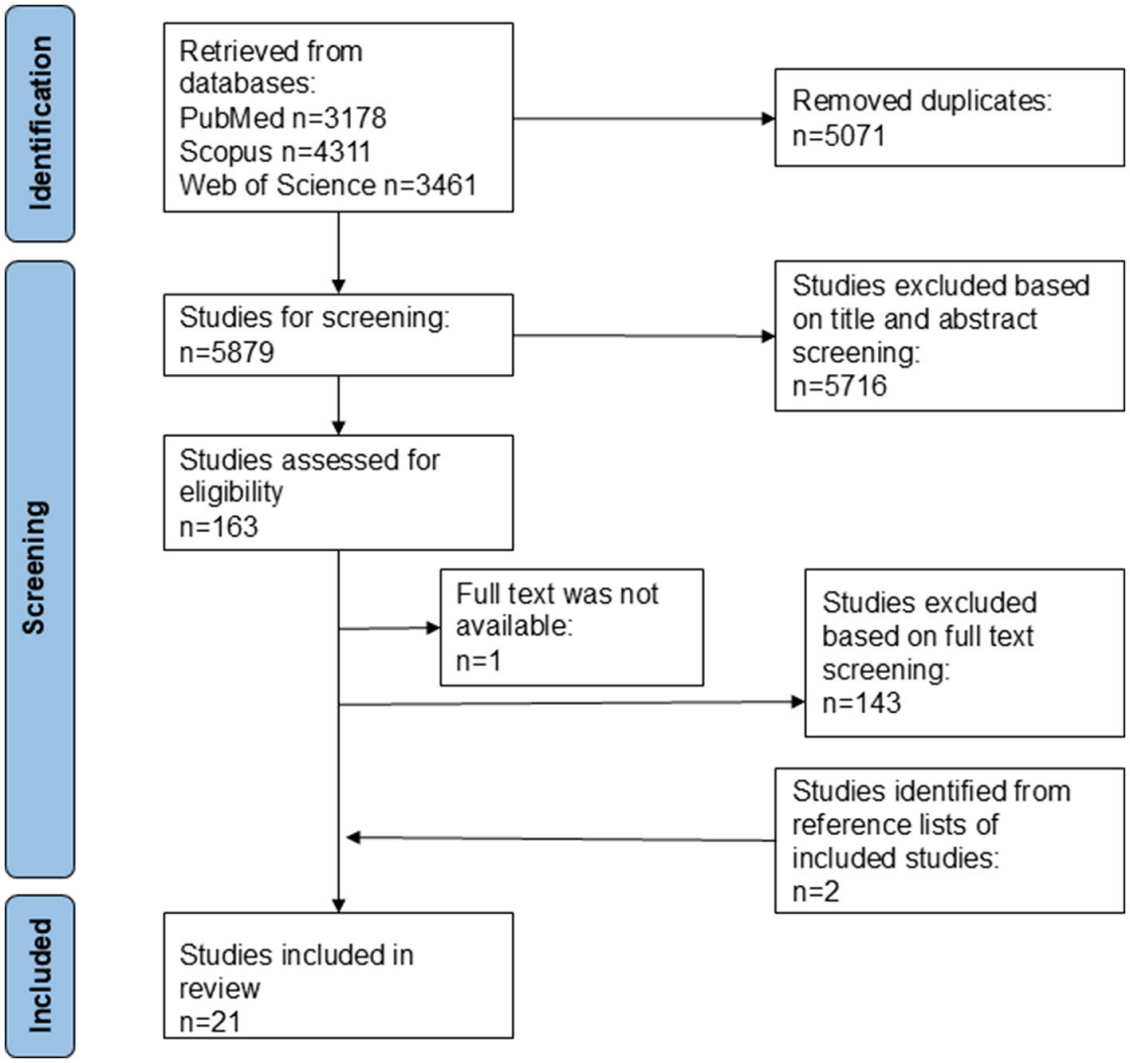
Study selection flow diagram.

We conducted a systematic literature search consisting of three central databases which were Scopus, PubMed, and Web of Science. The search included all publications from the beginning of the content provided by the databases until 9th of August 2022 (Figure 1). Grey literature was not included to our search because of possible lack of quality control, limited access to the data, bias and incompleteness of information, difficulties finding sources of information and inconsistent standards of publishing. Search terms were retrieved from the abstract, title, keywords, and Medical Subject Headings (MESH terms). Outcomes of interest: The outcomes of interest of our review were respiratory tract infections (both upper and lower) and pathogens (viral or bacterial) which can cause respiratory tract infections. Hence, we used the following search terms: respiratory infection, common cold, bronchitis, sinusitis, influenza, laryngitis, pharyngitis, pneumonia, otitis, adenovirus, rhinovirus, COVID-19 (see Supplement 3). Exposures of interest: The search terms representing exposure to weather were weather, temperature, wind, humidity, cold, hot, heat, season, precipitation, rain, climate, or sunlight. Confirming that the studies were conducted in urban environments we used the following search terms: urban, city, town, park, green space, and blue space. The full search strategies with the complete list of search terms are presented as Supplementary material. A reference list screening was performed for the full-text articles which met the inclusion criteria and were identified through the database searches.

The results from the literature search were processed by a screening and data extraction software platform Covidence (Covidence systematic review software, Veritas Health Innovation, Melbourne, Australia) which removed duplicates (Figure 1).

### Eligibility, outcome and exposure criteria

Studies that met the following a priori criteria were included: the study (i) was an original study of any relevant epidemiological study type (i.e., cohort, case-control, cross-sectional or ecological study), (ii) was written and published in English, (iii) reported any weather and urban environment parameters as exposures, (iv) reported respiratory tract infections as outcomes, (v) included a study population of any age and size, (vi) reported on the relations between weather and urban environment characteristics as exposures and respiratory tract infections as outcomes. Correspondingly, studies that did not meet one or more of the above-mentioned inclusion criteria (i–vi) were excluded.

### Title and abstract screening

The titles and abstracts of the studies were screened by one author (HH-P) to meet the inclusion criteria. From the identified articles we further decided to select studies that included any type of description connected to urban structure and function (such as population density, accessibility, mobility). Following the title and abstract screening, two authors (HH-P, TMI) evaluated independently the remaining 163 articles and agreed on the articles which met the inclusion criteria and were included in the full text review.

### Data extraction and quality assessment

Data was extracted from the included single studies to a structured word-form by the first author of the study. The data was later complemented by data from the other senior researchers. The full texts of the eligible studies were evaluated, and the main characteristics of these are presented in Table 1. This table includes reference information, study population, outcome of interest (RTIs), exposures of interest (weather variables and the determinants of urban environment), main results, and the score on the AXIS scale. The study quality was assessed using the AXIS Appraisal Tool for Cross-Sectional Studies (20) which includes 20 items. AXIS is developed to systematically assess the quality and risks of biases in cross-sectional studies, and includes the following domains: selection, exposure (risk factors), outcome, confounding, analysis and conflict of interest.

**Table 1:** Characteristics of included studies.

**Reference, study year**	**Study population and design**	**Outcome (RTI), period of data collection**	**Exposure (weather)**	**Exposure (urban environment)**	**Main results**	**The number of applicable answers to the questions of the AXIS tool (max 15)**
Ahmed et al. (2021)	24 countries and 70 cities/provinces around the world, register based ecological type study	COVID-19, the total number of infected cases and deaths (18.1.−24.4. 2020)	Average low and high temperatures, monthly average humidity	Population density	Temperature and average humidity ↑ or ↓: COVID-19 < - > Population density ↑: COVID-19 ↑	13
Coccia (2021)	55 cities in Italy, register based ecological type study	COVID-19, number of confirmed cases (1.3.−30.4. 2020)	Temperature, moisture, average wind speed	Population density	Temperature ↑: COVID-19 ↓ Wind ↓ and air pollution ↑: COVID-19 ↑ Population density ↑: COVID-19 ↑	10
Coşkun et al. (2021)	81 provinces in Turkey, register based ecological type study	COVID-19, cases (1.3.−31.3. 2020)	Temperature, rain, sunny days, wind speed	Population density	Temperature ↑ or ↓: COVID-19 < - > Humidity ↑ or ↓: COVID-19 < - > Wind ↑: COVID-19 ↑ Population density ↑: COVID-19 ↑	11
Dalziel et al. (2018)	603 cities in the United States, register based ecological type study	Influenza, weekly incidence (2002–2008)	Relative humidity	Population size	Amplitude of seasonal fluctuations in specific humidity ↑: epidemics intensity ↑ City size ↓: influenza season length ↓	10
De Angelis et al. (2021)	1,459 Lombardy municipalities, Italy, register based ecological type study	COVID-19, daily number of cases and excess of all-cause mortality (20.1.−16.4. 2020) and (1.3.−30.4. 2020)	Average winter temperature, average winter absolute humidity	Education level, private mobility use, distance to the closest hospital, beds in nursing homes, number of employees in bars, restaurants and mobile catering activities and employees in sports, entertainment and recreational activities	Temperature ↑: COVID-19 ↓ Absolute humidity ↑: COVID-19 ↑	15
Diao et al. (2021)	China (12 cities), England (20 cities), Germany (20 cities) and Japan (16 cities), register based ecological type study	COVID-19, spread duration and decay duration (1.1.−30.6. 2020)	Maximum and minimum temperature, Absolute humidity	total population and population density	Temperature ↑ or ↓: COVID-19 < - > Absolute humidity↑ or ↓: COVID-19 < - > Population density ↑: spread and duration of COVID-19 ↑	12
Halos et al. (2022)	Baghdad, Iraq and Kuwait City, Kuwait, register based ecological type study	COVID-19, morbidity and mortality (Feb-Dec 2020)	Hourly values of air temperature, relative humidity, wind speed	Population density	Temperature ↑: COVID-19 ↑ Relative humidity ↓: COVID-19 ↑ Population density ↑: COVID-19 ↑	10
Hassan et al. (2021)	Dhaka, Bangladesh, register based ecological type study	COVID-19, infection rate (1.5.−31.5. 2020)	Land surface temperature, rainfall, wind speed and pressure	Population density	Temperature ↑: COVID-19 ↓ Wind speed ↑: COVID-19 ↑ Population density ↑: COVID-19 ↑	12
Jamshidi et al. (2020)	USA (data at state and county levels), register based ecological type study	COVID-19, infections (1.1.−15.8. 2020)	The equivalent temperature (combined effect of temperature and humidity)	Urban density and population, mobility	Temperature ↑: COVID-19 ↓ Population ↑: COVID-19 ↑ Urban density ↑: COVID-19 ↑	13
Kodera et al. (2020)	14 prefectures in Japan, register based ecological type study	COVID-19, morbidity (confirmed infections) and mortality (deaths) rates (22.2.−25.5.2020)	Daily average, maximum and temperatures, daily average, maximum and minimum absolute humidity, daily average wind velocity	Population density, older adult population	Temperature ↑: COVID-19 ↓ Absolute humidity ↑: COVID-19 ↓ Population density ↑: COVID-19 ↑ Older adult percentage ↑: COVID-19 ↑	14
Kotsiou et al. (2021)	Milan, Rome, Naples, and Salerno in Italy, register based ecological type study	COVID-19, new confirmed infections (1.1.−8.4.2020)	Daily average PM10, PM2.5, daily average temperature, humidity (do not define whether relative of absolute humidity) and wind speed	Total population, population density, mean age of population	Temperature ↓ and PM10 ↑: COVID-19 ↑ Temperature ↓ and PM2.5 ↑: COVID-19 ↑ Population density ↑: COVID-19 ↑ Population's age ↑: COVID-19 ↑	12
Lin et al. (2022)	279 prefecture-level administrative regions in China, register based ecological type study	COVID-19, the growth rate of cumulative infections (1.1.−19.1.2020)	Diurnal mean temperature and relative humidity	Total population	Temperature ↑: COVID-19 ↑ until−7 degrees of Celsius and after that COVID-19 ↓ Relative humidity ↑: COVID-19 ↑ until 46 % and after that COVID-19 ↓ Population size ↑: COVID-19 ↑	12
Nakada and Urban (2021)	São Paulo, Brazil, register based ecological type study	COVID-19, infection rate (number of infected persons/days of infection) (24.3.−6.7.2020)	Daily temperature, relative humidity, wind speed, and ultraviolet radiation 3,7 and 14 days prior to infection	Total population, population density, social isolation rate	Temperature ↑: COVID-19 ↓ Relative humidity ↑ or ↓: COVID-19 < - > UV radiation ↑: COVID-19 ↓ Wind speed ↑: COVID-19 ↓ Population density ↑: COVID-19 ↑ Social isolation rate ↑: COVID-19 ↓	10
Pequeno et al. (2020)	27 Brazilian capital cities, register based ecological type study	COVID-19, daily cumulative counts of confirmed infections (26.2.−26.3.2020)	Mean daily temperature, relative humidity, solar radiation, precipitation (mm)	Population density, number of arriving flights	Temperature ↑: COVID-19 ↓ Relative humidity ↑ or ↓: COVID-19 < - > Solar radiation ↑ or ↓: COVID-19 < - > Population density ↑: COVID-19 ↑ Arriving flights ↑: COVID-19 ↑	12
Rader et al. (2020)	The prefectural level (*n* = 293) in China and at the province level (*n* = 108) in Italy, register based ecological type study	COVID-19 daily incidence, epidemic peakedness (1.12.−30.3.2020)	Daily temperature, relative humidity, atmospheric pressure	Total population, Population density, crowding index	Relative humidity ↑: COVID-19 ↓ Population density ↑: COVID-19 ↑	12
Rashed et al. (2020)	16 prefectures in Japan, register based ecological type study	COVID-19, spread and decay durations (15.3.−25.3.2020)	Daily average, maximal, minimal temperature and absolute humidity	Population density	Temperature ↑: COVID-19 ↓ Absolute humidity ↑: COVID-19 ↓ Population density ↑: COVID-19 ↑	14
Rubin et al. (2020)	211 counties, United States, register based ecological type study	COVID-19, daily incident infection counts (25.2.−23.4.2020)	Wet bulb temperature	Population density	Temperature ↑: COVID-19 ↓ until 11 degrees of Celsius COVID-19 ↑ and after 20 degrees again COVID-19 ↓ Population density ↑: COVID-19 ↑	14
Salcido and Castro (2022)	Mexico City Metropolitan Area, register based ecological type study	COVID-19, daily confirmed infections (1.2.−31.12.2020)	Hourly temperature, relative humidity, wind speed, wind direction	Population density	Temperature ↑: COVID-19 ↓ Relative humidity ↑: COVID-19 ↓ Wind ↑: COVID-19 ↑ Population density ↑: COVID-19 intensity ↓	12
Yang et al. (2021)	324 cities, China, register based ecological type study	COVID-19, confirmed infections and deaths (5 days) (29.1.−29.2.2020)	Average (five days) temperature, relative humidity, solar radiation intensity	City size, gross domestic product (GDP), cultural radiation area and urban built-up area	Temperature ↑ or ↓: COVID-19 < - > Relative humidity ↑ or ↓: COVID-19 < - > Solar radiation ↑ or ↓: COVID-19 < - > City size ↑: COVID-19 ↑	11
You and Pan (2020)	989 counties, United States, register based ecological type study	COVID-19, daily confirmed infections (20.1.−13.3.2020 and 14.3.−24.5.2020)	Daily temperature	Population density, urban vegetation cover	Temperature ↑: COVID-19 ↓ Population density ↑: COVID-19 ↑ Urban vegetation ↑: COVID-19 ↓	12
Zhang et al. (2021)	650 communities in Wuhan, China, register based and own data collection, ecological type study	COVID-19, confirmed infections (period of data collection uncertain)	Temperature, humidity (does not define whether relative or absolute humidity)	Visible Green Index, population density, the number of houses, the number of buildings and the cost of property in communities	Temperature ↑: COVID-19 cases ↓ and COVID-19 recovery cases ↑ Humidity ↑: COVID-19 ↑ Visible green index ↑: COVID-19 ↓	11

< - >, no change; ↑, increase of the exposure or the outcome; ↓, decrease of the exposure or the outcome. AXIS, Appraisal tool for Cross-Sectional Studies, score 0–15.

The authors of this review (HH-P, TTH) agreed that five questions from the AXIS tool were not applicable for the included studies (questions 7, 13, 14, 15 and 20, Supplement 4). These questions were related to whether non-response bias was discussed and addressed in the studies. These were not applicable in the studies which used register-based data. In addition, some of the study designs (such as non-invasive ecological studies) did not allow us to evaluate questions related to ethical approval and consent of participants. The questions can be answered either “yes” (14 questions from the 15 included questions) or “no” (question number 19). Each applicable answer (yes or no) to the question of the AXIS tool indicates that this factor connected to the quality of the study has been considered in the study. The greater the number of questions that have been answered appropriately, the higher the quality of the research. When unfit questions were excluded the number of applicable answers describing the quality of the study may vary between 0 and 15. Two authors (HH-P, TTH) independently evaluated the quality of each study, and disagreements were discussed and resolved.

The data of the study was stored at a secure platform at the University of Oulu and its Research Unit of Population Health, Finland.

We synthesized the evidence of our systematic review in the discussion through a narrative synthesis. This involves summarizing the results in a descriptive manner, discussing trends, inconsistencies, and potential reasons for variability of the results.

## Results

### Study selection and characteristics of the studies

The search strategy identified 3,178 articles from PubMed, 4,311 articles from Scopus, and 3,461 from Web of Science. The flow chart of literature search is presented in Figure 1. After the Covidence excluded duplicates, 5879 articles remained for screening. Following the title and abstract screening full texts of 163 articles were further evaluated for inclusion. These articles' references were also examined after which two additional studies were identified to be further evaluated. After the full text evaluation 21 articles were included in the qualitative assessment.

Data concerning exposure and/or outcome were collected from different national and worldwide registers, databases, satellite data, official reports and statistics, and thus, represented ecological study designs. One of the selected studies involved research related to influenza (21) and 20 studies examined the occurrence of COVID-19. Our search did not identify studies representing lower RTIs or bacterial infections. Concerning weather as a measure of exposure, 19 of the selected studies presented information on temperature, while humidity was reported in 15, wind speed in 5 and ultraviolet radiation in 3 studies. Often weather was defined as daily measures, but the period of exposure varied between hourly to even monthly measures. All studies presented population density as a descriptive variable for urban areas. In summary, the included studies examined the relationship between population density and respiratory tract infections, but we did not find any research which studied the association between urban built environment, weather, and infectious diseases.

### Quality assessment of the studies

According to the quality assessments, the selected studies were of relatively high quality. The number of suitable answers describing the quality of the research varied from 10 to 15 and where 15 applicable answers indicate fulfilling all quality criteria (Table 1). In general, lower quality studies lacked information on how the statistical significance was determined, the conclusions were not supported by the results and the discussion of study limitations was missing (Supplement 4).

### Weather variables and RTIs

The observed associations between weather and RTIs in urban environments are presented in Table 1.

Nineteen studies assessed the association between temperature and RTIs. Eleven of the included studies found that higher ambient temperature was associated with a decrease in the incidence of RTIs in the studied populations (11, 22–31). Four studies did not find an association between ambient temperature and COVID-19 (32–35). Only one study found a positive correlation between temperature and infections (36), while two studies reported mixed results (37, 38). One study showed that higher temperature was associated with a shorter duration of an epidemic in an urban area (39).

Fourteen studies assessed the association between humidity and RTIs. Low humidity was associated with the occurrence of infections in five studies (25, 29, 36, 39, 40). High humidity increased the occurrence of infections in three studies (21, 30, 36). Furthermore, one of these identified a threshold of relative humidity being 46% where after a positive association became a negative association (37). The remaining six studies did not find an association between humidity and RTIs (27, 28, 32–35).

Five studies assessed the association between wind speed and RTIs. Three of these reported a higher occurrence of COVID-19 infections with higher wind speeds (23, 29, 33), whereas two of the studies showed an inverse association between wind speed and RTIs (11, 27).

### Urban built environment and RTIs

Fifteen studies examined the association between population density and RTIs. Fourteen of the studies found that cities with higher population density had higher risk of respiratory infections than those of lower population density (11, 23–28, 30, 32, 33, 36, 38–40). Only one study demonstrated a lower intensity of infections in cities with higher than lower population density (29).

City or population size were analyzed in two studies which concluded that there were more infections in larger cities with higher population size than in smaller cities with lower population sizes (35, 37). In addition, smaller cities involved a shorter infection period than larger cities (21). One study from the USA reported that the increase of urban vegetation decreased cumulative infection rate of COVID-19 (30).

## Discussion

To our knowledge, this is the first systematic review studying the potential association between urban built environment, weather and RTIs. Our results show that low temperature in urban areas was related to an increased risk of RTIs. Humidity was also related to the occurrence of RTIs, but the results were inconclusive. The association between other weather parameters (wind and solar radiation) and the risk of RTIs showed inconsistent findings. Most of the studies included population density as an indicator of urban environment and showed that high population density is associated with increased risk of RTIs. This review did not find studies where the association between urban built environment features, and their potential influence on weather and consequently to the occurrence of RTIs had been examined.

### Validity of the results

The strengths of our study include selecting individual studies based on a clearly defined search strategy. We used broad search terms to be able to identify as many suitable urban studies (including features of urban design) as possible. In addition, primary PubMed, Scopus and Web of Science searches, were complemented using references cited by the articles identified in the primary search. Using predefined search criteria and multiple databases helps reduce selection bias. In order to minimize reviewer bias, two authors evaluated independently the eligibility of the studies according to a priori set inclusion and exclusion criteria. Any disagreements were settled by discussion. The validated and widely used AXIS appraisal tool was used to assess the quality of the studies (41–44).

In general, our quality assessment based on peer-evaluation showed that the selected studies were often carried out according to relatively good research standards (number of applicable answers range 10–15). Despite the seemingly accurate reporting of the studies included, there are limitations related to these that could affect the interpretation of the results. The empirical evidence related to the studies included is limited by the fact that most of them were cross-sectional by design. Thus, it is not possible to judge the temporal relation between exposure and the outcome of interest. All the studies were ecological studies creating a possibility of ecological fallacy and where broad population level assessment of exposure and outcome do not necessarily apply to a higher degree of spatial (district or neighborhood) resolution or individual level. In addition, the differing national pandemic responses and restrictions to human mobility likely affected exposure and consequently the occurrence of infections. The studies utilized data obtained from various registers which coverage and reliability may vary. Some of the studies applied only correlation analyses that do not capture the effects of multiple co-occurring exposures (e.g., temperature and air pollution) or confounding factors (e.g., population density and demographic, socioeconomic factors) potentially affecting the association between environmental exposures and the occurrence of infections. Except for two studies (24, 38) which included combined measures of temperature and humidity as exposure, the issue of multicollinearity between the weather parameters was not addressed. Also, the association between weather and infections is often non-linear and which was seldom (38) considered in the studies included. It should also be noted that the studies involved various geographic areas and where the mode of transmission of respiratory infections could vary between different climatic zones and be differentially affected by weather (7). A limitation of our study is that we did not find studies from Africa and Oceania. The RTIs of the studies were, except for one study (21), limited to COVID-19 infections. Empirical evidence of different respiratory pathogens could have improved the interpretation of our findings. Finally, the language of the studies was limited to English which might have precluded identification of relevant studies. Irrespective of our rigorous methodology, we acknowledge the possibility of some selection bias (i.e., non-representative study populations or study designs, geographical bias, language bias). Also, publication bias could have occurred when research with significant or positive findings is more likely to be published, while studies with null or negative findings are underreported or unpublished. It is also possible that the studies included in the systematic review could not consider all potential confounding factors that could influence the relationship between exposure and outcome. It should also be noted that this review did not include studies related to indoor exposure where likely most person-to-person transmissions occur (7), and which could provide complementary information when examining the association between urban exposures and the occurrence of infections.

### Temperature and humidity

This review showed that low temperature is often associated with increased risk of RTIs in urban areas (11, 22–30, 38, 45). These findings are consistent with previous research concerning COVID-19, as well as other pathogens causing RTIs. A retrospective study reported that low temperature and humidity were associated with higher influenza mortality in New York (46). Qi et al. (47) showed that the occurrence of influenza activity was related to low temperature in Chongqing, China. Also, hospitalizations due to RSV infections among children have been shown to occur more frequently at low temperatures in Canada (48) or subtropical China (49). In addition, a Finnish study showed that COVID-19 incidence increased at low temperatures but indicated a need for a longer follow-up to confirm a seasonal pattern (50). Studies outside the urban settings have also shown that lowered temperature and humidity increase the occurrence of RTIs during military training (51, 52). The rest of the studies in this review found either no association (32–35), a positive association (36) or mixed observations (37, 38) when examining the relationship between temperature and COVID-19 infections. The somewhat contradictory findings can be related to varying climates and populations of the studied cities, reflect differences in their urban design, such as are they built as tight blocks or are they walkable, mixed-use neighborhoods, or be due to the employed study designs and methodological approaches (see discussion related to validity of the results). The differing findings could also indicate that there is a specific environmental temperature range for each respiratory pathogen where its viability and capability to cause an infection is optimal (7). In summary, many of the studies included in this review confirm earlier findings suggest that low temperature increases the occurrence of infections (7).

Environmental humidity (absolute or relative) was associated with the risk of RTIs in several of the included studies. These studies presented divergent findings where either low (25, 29, 36, 39, 40) or high humidity (22, 31, 37) was associated with the risk of RTIs. The rest of the studies (27, 28, 32–35) did not find an association between humidity and the risk of RTIs. Previous findings in urban areas at a global level have shown that low absolute humidity was associated with higher incidence of COVID-19 infections (8). Similarly, the relative risk of influenza-like-illness increased with lowered absolute humidity in a Chinese municipality (47). It should be noted that humidity is strongly interlinked with temperature, and which was not considered in most of the included studies. Only two studies (24, 38) used exposure measures that combined temperature and humidity (wet bulb temperature, equivalent temperature) and examined their relation to infections. Rader et al. (40) found a non-linear relationship and where both low and high wet bulb temperature increased the occurrence of COVID-19 infections. Instead, Jamshidi et al. (24) suggested that weather was a non-influential factor for COVID-19 infections but recommended future studies to be conducted at a finer-scale accounting for urban form, function, and density.

There are a few potential explanations for the associations between temperature, humidity, and RTIs. Respiratory viruses can remain stable and infectious as aerosols in the cold for extended periods increasing the likelihood of transmission (6–8). In addition, inhaling both cold and dry air can alter the functional properties of the mucous membranes in the respiratory tract, including the nasal epithelium, which can increase the susceptibility of the host to infections (53, 54). Some respiratory viruses also tend to be more prevalent during the winter months, contributing to the seasonal occurrence of RTIs (7, 55, 56). Seasonality of RTIs could also be influenced by different host factors, such as altering immunity of humans between winter and summer months (6). Finally, environmental temperature could also influence human outdoor activities (such as spending more time indoors in winter), and this contributes to the risk of contracting an infection (38).

The studies included in this review did not specifically assess how the built environment of cities influenced the observed association between temperature and RTIs. Urban structures could affect exposure to temperature and humidity in cities and create, in terms of weather, spatially heterogenous districts and neighborhoods. For example, building materials can absorb or release heat acting as heat sinks during the daytime and as heat sources during the nighttime (57). The orientation of the buildings can reduce or increase the heat load. Buildings facing the south maximize exposure to solar radiation, contributing to higher temperatures (58). Lack of green spaces in cities reduce evaporative cooling and contribute to accelerating the UHI effect in cities (59). The proliferation of impervious surfaces, such as roads and pavements traditionally covered with asphalt reduces the cooling effect of evaporation, contributing to higher temperatures in urban areas (60). Closely spaced buildings can trap heat and reduce natural ventilation which contributes to higher temperatures within cities (61). Finally, different anthropogenic activities, such as industrial processes, heating of houses and traffic further contribute to warming of urban areas.

### Wind

Wind can alter the occurrence of RTIs in cities through its influence on the transmission and spread of respiratory pathogens. The studies included in our review showed that either low (11) or high (23, 29, 33) wind speed in cities increased the occurrence of RTIs. Though, high wind speed could also reduce (27) or have no influence on the occurrence of RTIs (25). A recent study suggested that low wind speed was associated with increased influenza activity (47). The reason for the deviating findings is likely due to different environmental conditions, study designs and analytical approaches. For example, wind speed is a weather variable which can show substantial temporal and spatial variation (62, 63). It could vary significantly within cities, as well as between different urban neighborhoods and regions (62, 64, 65).

Wind could affect the occurrence of RTIs through a few mechanisms. Firstly, it can disperse respiratory droplets more quickly over long distances (66). This increases the potential for these infectious particles to spread and cause RTIs. On the other hand, wind can enhance outdoor air circulation, which could dilute and decrease the number of fine and coarse aerosols in the air (67). However, there is little evidence that this would lead to lower microbial exposure and further to lower risk of respiratory tract infection in outdoor settings (11). Wind can also bring contaminated air from one area to another, potentially increasing exposure risks (67). Finally, increased wind speed may also decrease air pollution within the cities, lower the burden on the airways and further reduce the occurrence of COVID-19, for example (68).

The studies included in the review did not specifically assess how the structures of cities influenced the observed association between wind and RTIs. Exposure to wind of urban areas can be influenced by the density, height or length, location and even shapes of buildings in cities (17). Tall and closely spaced buildings can obstruct the flow of wind, creating wind shadows and reducing wind speeds at street level (69). Buildings can also generate turbulence, causing unpredictable gusty wind patterns and local variations in wind intensity (70, 71). Streets and other open spaces can further affect the direction and speed of wind, allowing it to move more freely (72, 73). Also, the higher temperatures of urban heat islands can create vertical temperature gradients that influence the mixing of air and affect wind patterns (74).

### Population density and other urban environment characteristics

Our results showed that the population density was the most studied urban characteristic of interest in the selected studies. The main observation was that cities with higher population density had more RTIs than those with lower population density irrespective of the city size.

In densely populated areas, people are more likely to be in close connection to one another, increasing the potential for respiratory pathogens to spread from person to person mainly through airborne transmission (75). These increased contacts can lead to higher transmission rates, especially in crowded indoor spaces. Dasgupta et al. (76) observed that urban areas with higher density of housing units per structure and crowded housing units (i.e., more persons than rooms) were more likely to become COVID-19 hotspots in U.S. Similarly, Rader et al. (40) suggested that high urban density and crowding could lead to sustained and longer outbreaks of infection epidemics. Spread of RTIs is not only a question of the challenge related to the characteristics and internal dynamics of cities, but also of the connections between cities and the mobility of people. Densely populated urban areas often have extensive public transportation systems, which can serve as national and international hubs for infection transmission as people are in close contact on buses, subways, trains, and airplanes (77–79). This also allows the pathogens to move quickly within or between the cities (79, 80). In a Japanese study, the use of public transportation and the time spent commuting increased the risk of COVID-19 infection (81). Overall, the association between population density or neighborhood design and transmission of RTIs have shown contradictory findings and more consistent measures and methods are needed to confirm their possible relations (82, 83).

Environmental greenness was shown to relate to RTIs in two studies. A study from the USA showed that each 1% increase in the percentage of urban vegetation led to a 2.6% decrease in cumulative cases of COVID-19 (30). In contrast, the other study used a machine-learning-based visible green-index (VGI) which was positively related to COVID-19 infections (31). The few recent studies examining the association between environmental greenness and RTIs have shown a reduced number of infections with increasing levels or availability of greenness (84, 85).

### Summary of our findings

**Figure 2:**
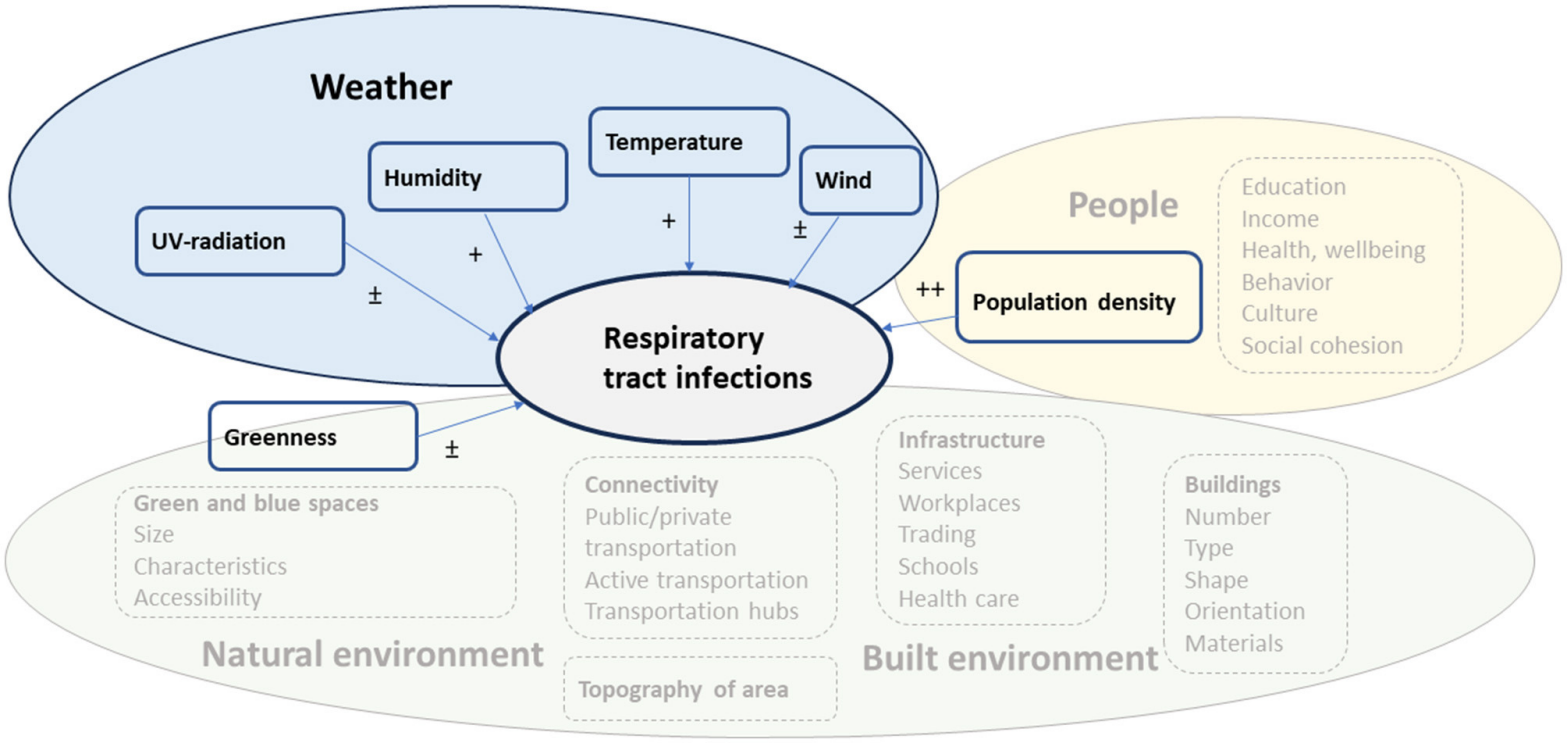
Suggested framework for environmental factors influencing the occurrence of RTIs. The factors included in the systematic review are presented as boxes with a solid line and those from which empirical findings are lacking in the systematic review as boxes with a dashed line. The level of evidence is presented as ++ (several studies showing consistent findings), + (a few studies showing consistent findings), ± (studies show inconsistent results).

We have summarized our results and suggested a framework of the environmental factors influencing the occurrence of RTIs in Figure 2. The empirical evidence obtained from our review suggests that population density was strongly connected to the occurrence of RTIs. Related to this, human contacts (and risk of RTIs) are influenced by various characteristics of the urban residents (socioeconomic factors, health, wellbeing, behavior, culture and social belonging). Furthermore, we observed that weather, and especially temperature and humidity, are associated with RTIs. Though, the direction of the association between humidity and infections varied. Likewise, exposure to wind or UV-radiation showed weak and inconsistent associations with RTIs (82, 86).

Our findings suggest that both climate and population density affect the spread and occurrence of RTIs, and which agrees with modeling presented by Spada (87). Based on previous empirical evidence related to urban design and health various factors related to the built and natural environment can either directly or indirectly influence the occurrence of RTIs, but for which empirical evidence is largely lacking. We only observed a weak association between environmental greenness and RTIs and further studies are needed to examine whether size, characteristics and accessibility to green or blue spaces influence the occurrence of RTIs.

## Conclusions

The results of this review show that exposure to certain weather conditions influences the risk of contracting RTIs in urban areas. Especially low temperature was associated with a higher risk for contracting an RTI. Also, humidity was linked with RTIs, but the direction of this relation varied. Because many of the weather parameters are interlinked, exposure related to the occurrence of RTIs should be examined in future studies as a composition of multiple meteorological conditions. Most of the recent studies investigated weather parameters in relation to COVID-19 and where research related to other respiratory pathogens would provide complementary information. Our study also shows that the association between urban built environment, weather and RTIs has not been studied. Only population density, which represents an indirect measure of urban design, was shown to be positively associated with COVID-19 infections.

Based on earlier empirical evidence we suggest that the built environment could directly affect the occurrence of RTIs, but also indirectly through altered city or neighborhood-level exposures to weather. Firstly, compact cities (but avoiding overcrowding) involving mixed and decentralized land use reduce the need for transport and affect the risk of infections. Sufficient access to health services and promoting physical activity improve public health but are also keys for successful management of epidemics or pandemics (82). Planning walkable and cyclable cities could contribute to reducing air pollution, noise and urban heat island effects (86) and, at the same time, reduce infections due to use of public transport (82, 88). Urban design should include enough accessible green spaces which help alleviate urban heat strain (15) and reduce air pollution and indirectly also the occurrence of infections (89, 90). Though, related empirical evidence is lacking. Urban planning should also focus on using reflective building materials or colors to reduce heat-related impacts. Though, their impact on respiratory infections remains uncertain. The location and direction of buildings and streets should allow free flow of air which reduces air pollution but could also remove respiratory pathogens.

Future studies need to investigate how urban structures modify exposure to weather and air pollution, and consequently, affect contracting an infection. First, this requires collecting longitudinal data involving high spatial resolution and assessing how weather and air pollution is affected by, for example, buildings, streets and parks. After mapping the likely spatially heterogeneous exposures across different urban districts or neighborhoods this data could be combined with observed RTIs according to residential address of the infected persons. This type of empirical research could provide concrete support for urban planners.

## Data Availability

The original contributions presented in the study are included in the article/Supplementary material, further inquiries can be directed to the corresponding author.
