# Supplementary material for: The influence of weather and urban environment characteristics on upper respiratory tract infections: a systematic review
Source: Front Public Health. 2025 Feb 10;13:1487125. doi: 10.3389/fpubh.2025.1487125 (PMC11849499; doi:10.3389/fpubh.2025.1487125)
Supplement: Supplementary file 3 [file Table_3.docx]

**Supplement**

**Search terms and used databases of the systematic review**

Scopus:

( TITLE-ABS-KEY (weather OR temperature* OR wind* OR humid* OR cold OR hot OR heat OR season* OR precipitation OR rain* OR climat* OR sunlight) AND TITLE-ABS-KEY (urban OR city OR cities OR town* OR park OR parks OR "green space*" OR "blue space*") AND TITLE-ABS-KEY ("respiratory infection*" OR "Respiratory Tract Infection*" OR bronchitis OR sinusitis OR "common cold" OR influenza OR laryngitis OR pharyngitis OR pneumonia OR otitis OR "covid-19" OR "Severe Acute Respiratory Syndrome" OR adenovirus* OR rhinovirus*)) AND (LIMIT-TO (DOCTYPE, "ar") OR LIMIT-TO (DOCTYPE, "re")) AND (LIMIT-TO (LANGUAGE, "English"))

PubMed:

((((("Weather"[Mesh]) OR "Climate"[Mesh])) OR (weather[Text Word] OR temperature*[Text Word] OR wind*[Text Word] OR humid*[Text Word] OR cold[Text Word] OR hot[Text Word] OR heat[Text Word] OR season*[Text Word] OR precipitation[Text Word] OR rain*[Text Word] OR climat*[Text Word] OR sunlight[Text Word])) AND ((((((("Urban Health"[Mesh]) OR "Urban Health Services"[Mesh]) OR "Urban Population"[Mesh]) OR "City Planning"[Mesh]) OR "Cities"[Mesh]) OR "Parks, Recreational"[Mesh]) OR (urban[Text Word] OR city[Text Word] OR cities[Text Word] OR town*[Text Word] OR park[Text Word] OR parks[Text Word] OR "green space*"[Text Word] OR "blue space*"[Text Word]))) AND (((((((((((("Respiratory Tract Infections"[Mesh:NoExp]) OR "Bronchitis"[Mesh]) OR "Common Cold"[Mesh]) OR "Influenza, Human"[Mesh]) OR "Laryngitis"[Mesh]) OR "Pharyngitis"[Mesh]) OR "Pneumonia"[Mesh]) OR "Otitis"[Mesh]) OR "Severe Acute Respiratory Syndrome"[Mesh]) OR "Adenovirus Infections, Human"[Mesh]) OR "Rhinovirus"[Mesh]) OR ("respiratory infection*"[Text Word] OR "Respiratory Tract Infection*"[Text Word] OR bronchitis[Text Word] OR sinusitis[Text Word] OR "common cold"[Text Word] OR influenza[Text Word] OR laryngitis[Text Word] OR pharyngitis[Text Word] OR pneumonia[Text Word] OR otitis[Text Word] OR "covid-19"[Text Word] OR "Severe Acute Respiratory Syndrome"[Text Word] OR adenovirus*[Text Word] OR rhinovirus*[Text Word]))

Web of Science

weather OR temperature* OR wind* OR humid* OR cold OR hot OR heat OR season* OR precipitation OR rain* OR climat* OR sunlight (Topic) AND urban OR city OR cities OR town* OR park OR parks OR "green space*" OR "blue space*" (Topic) AND "respiratory infection*" OR "Respiratory Tract Infection*" OR bronchitis OR sinusitis OR "common cold" OR influenza OR laryngitis OR pharyngitis OR pneumonia OR otitis OR "covid-19" OR "Severe Acute Respiratory Syndrome" OR adenovirus* OR rhinovirus* (Topic) and Article or Review Article (Document Types) and English (Languages)
