# Supplementary material for: The influence of weather and urban environment characteristics on upper respiratory tract infections: a systematic review
Source: Front Public Health. 2025 Feb 10;13:1487125. doi: 10.3389/fpubh.2025.1487125 (PMC11849499; doi:10.3389/fpubh.2025.1487125)
Supplement: Supplementary file 4 [file Table_4.docx]

|  | Intro | Methods | | | | | | | | | | Results | | | | | Discussion | | Other | | Total score (max 15) |
| --- | --- | --- | --- | --- | --- | --- | --- | --- | --- | --- | --- | --- | --- | --- | --- | --- | --- | --- | --- | --- | --- |
|  | 1. | 2. | 3. | 4. | 5. | 6. | 7. | 8. | 9. | 10. | 11. | 12. | 13. | 14. | 15. | 16. | 17. | 18. | 19. | 20. |  |
| Ahmed 2021 | Y | Y | Y | Y | Y | Y | N/A | Y | N | Y | Y | Y | N/A | N/A | N/A | Y | Y | N | N | N/A | 13 |
| Coccia 2021 | Y | Y | Y | Y | N | Y | N/A | Y | Y | N | Y | N | N/A | N/A | N/A | Y | N | N | N | N/A | 10 |
| Coskun 2021 | Y | Y | Y | Y | N | Y | N/A | Y | Y | Y | N | N | N/A | N/A | N/A | Y | N | Y | N | N/A | 11 |
| Dalziel 2018 | Y | Y | Y | Y | N | Y | N/A | Y | Y | N | Y | N | N/A | N/A | N/A | Y | N | N | N | N/A | 10 |
| De Angelis 2021 | Y | Y | Y | Y | Y | Y | N/A | Y | Y | Y | Y | Y | N/A | N/A | N/A | Y | Y | Y | N | N/A | 15 |
| Diao 2021 | Y | Y | Y | Y | N | Y | N/A | Y | Y | N | Y | Y | N/A | N/A | N/A | Y | N | Y | N | N/A | 12 |
| Halos 2022 | Y | Y | Y | Y | N | Y | N/A | Y | N | N | Y | Y | N/A | N/A | N/A | Y | N | N | N | N/A | 10 |
| Hassan 2021 | Y | Y | Y | Y | Y | Y | N/A | Y | Y | N | Y | Y | N/A | N/A | N/A | Y | N | Y | Y | N/A | 12 |
| Jamshidi 2020 | Y | Y | Y | Y | N | Y | N/A | Y | Y | N | Y | Y | N/A | N/A | N/A | Y | Y | Y | N | N/A | 13 |
| Kodera 2020 | Y | Y | Y | Y | Y | Y | N/A | Y | Y | Y | Y | Y | N/A | N/A | N/A | Y | N | Y | N | N/A | 14 |
| Kotsiou 2021 | Y | Y | Y | Y | N | Y | N/A | Y | Y | N | Y | Y | N/A | N/A | N/A | Y | N | Y | N | N/A | 12 |
| Lin 2022 | Y | Y | Y | Y | Y | Y | N/A | Y | Y | N | Y | Y | N/A | N/A | N/A | Y | N | N | N | N/A | 12 |
| Nakada 2021 | Y | Y | Y | Y | N | Y | N/A | Y | Y | N | Y | Y | N/A | N/A | N/A | Y | N | N | Y | N/A | 10 |
| Pequeno 2020 | Y | Y | Y | Y | N | Y | N/A | Y | Y | N | Y | Y | N/A | N/A | N/A | Y | N | Y | N | N/A | 12 |
| Rader 2020 | Y | Y | Y | Y | N | Y | N/A | Y | Y | N | Y | Y | N/A | N/A | N/A | Y | N | Y | N | N/A | 12 |
| Rashed 2020 | Y | Y | Y | Y | N | Y | N/A | Y | Y | Y | Y | Y | N/A | N/A | N/A | Y | Y | Y | N | N/A | 14 |
| Rubin 2020 | Y | Y | Y | Y | N | Y | N/A | Y | Y | Y | Y | Y | N/A | N/A | N/A | Y | Y | Y | N | N/A | 14 |
| Salcido 2022 | Y | Y | Y | Y | Y | Y | N/A | Y | Y | N | N | Y | N/A | N/A | N/A | Y | N | Y | N | N/A | 12 |
| Tchicaya 2021 | Y | Y | Y | Y | Y | Y | N/A | Y | Y | Y | Y | Y | N/A | N/A | N/A | Y | Y | Y | N | N/A | 15 |
| Yang 2021 | Y | Y | Y | Y | Y | Y | N/A | Y | Y | N | Y | Y | N/A | N/A | N/A | Y | N | N | Y | N/A | 11 |
| You 2020 | Y | Y | Y | Y | Y | Y | N/A | Y | Y | N | Y | Y | N/A | N/A | N/A | Y | N | Y | Y | N/A | 12 |
| Zhang 2021 | Y | Y | Y | Y | N | Y | N/A | Y | N | N | Y | Y | N/A | N/A | N/A | Y | N | Y | N | N/A | 11 |
|  | 1. | 2. | 3. | 4. | 5. | 6. | 7. | 8. | 9. | 10. | 11. | 12. | 13. | 14. | 15. | 16. | 17. | 18. | 19. | 20. |  |

1. Were the aims/objectives of the study clear?
2. Was the study design appropriate for the stated aim(s)?
3. Was the sample size justified?
4. Was the target/reference population clearly defined? (Is it clear who the research was about?)
5. Was the sample frame taken from an appropriate population base so that it closely represented the target/reference population under investigation?
6. Was the selection process likely to select subjects/participants that were representative of the target/reference population under investigation?
7. Were measures undertaken to address and categorise non-responders? NA
8. Were the risk factor and outcome variables measured appropriate to the aims of the study?
9. Were the risk factor and outcome variables measured correctly using instruments/measurements that had been trialled, piloted or published previously?
10. Is it clear what was used to determined statistical significance and/or precision estimates? (e.g. p-values, confidence intervals)
11. Were the methods (including statistical methods) sufficiently described to enable them to be repeated?
12. Were the basic data adequately described?
13. Does the response rate raise concerns about non-response bias? NA
14. If appropriate, was information about non-responders described? NA
15. Were the results internally consistent? NA
16. Were the results presented for all the analyses described in the methods?
17. Were the authors' discussions and conclusions justified by the results?
18. Were the limitations of the study discussed?
19. Were there any funding sources or conflicts of interest that may affect the authors’ interpretation of the results?
20. Was ethical approval or consent of participants attained? / Was there statement if the ethical approval was not required because of register data? NA
